# Anticancer effect of black tea extract in human cancer cell lines

**DOI:** 10.1186/s40064-015-0871-4

**Published:** 2015-03-14

**Authors:** Katarína Koňariková, Miriam Ježovičová, Ján Keresteš, Helena Gbelcová, Zdeňka Ďuračková, Ingrid Žitňanová

**Affiliations:** Institute of Medical Chemistry, Biochemistry and Clinical Biochemistry, Faculty of Medicine, Comenius University, Sasinkova 2, 811 08 Bratislava, Slovak Republic; Molecule of Life, Ltd, Lichnerova 38, 903 01 Senec, Slovakia; Institute of Medical Biology, Genetics and Clinical Genetics, Comenius University, Sasinkova 4, 811 08 Bratislava, Slovak Republic

**Keywords:** *Camellia sinensis*, Black tea, Cancer, Protective effect, Apoptosis

## Abstract

In this study we investigated effects of natural extract from the black tea *Camellia sinensis* (BTE) against human colon carcinoma cell line HT-29, human breast carcinoma cell line MCF-7, human alveolar carcinoma cell line A549 and healthy cell line NIH-3T3. We identified concentration range for cytotoxic/antiproliferative effects using MTT assay and the trypan blue assay, gel electrophoresis we employed to determine the type of cell death induced by BTE and DNA damage we determined by comet assay. Different concentrations of the extract (0.00078 - 5 μg/mL) we added to the cultured cells and incubated for 216 h. BTE showed cytotoxic effects against all carcinoma cell lines, however HT-29 and MCF-7 cells were more sensitive than A549. BTE showed no antiproliferative effect against healthy cells NIH-3T3 at tested concentrations. We found no apoptotic cell death in HT-29 and MCF-7 cells after 72 h of incubation in case of single administration of BTE but in case of repetitive administration of BTE (BTE was added to the cells each day) we found apoptotic cell death in HT-29 after 72 h incubation. BTE induced also DNA strand breaks and oxidative damage to DNA in carcinoma cells HT-29 and MCF-7.

## Introduction

Black tea has a long history of use dating back to China approximately 5,000 years ago. It is made from the dried leaves of *Camellia sinensis*, a perennial evergreen shrub formerly known as *Thea sinensis*. It is native to southeastern Asia. Green tea, black tea, and oolong tea are all derived from the same plant. Black tea results from the oxidation of *Camellia sinensis* leaves. The chemical components in tea include alkaloids (theobromine, caffeine, theophylline), polyphenols, amino acids, polysaccharides, volatile acids, vitamins, lipids as well as inorganic elements (Liang et al. 2013; Xiaorong et al. 2015; Scoparo et al. 2014). Black tea is used for treating headaches, low blood pressure, preventing heart disease, including atherosclerosis and heart attack, preventing Parkinson’s disease, reducing the risk of stomach and colon cancer, lung, ovarian and breast cancers (Lee and Foo 2013). The aim of our study was to evaluate the potential anticancerogenic effect of the black tea extract (BTE) on different types of carcinoma cell lines.

## Results

### Antiproliferative effects of BTE - single administration of BTE

BTE showed the highest cytotoxic effect against HT-29 (human colon carcinoma cell line) cells after 24 h of influence (Table 1). IC_50_ was lower than the lowest concentration used (0.00078 μg/mL) and stayed unchanged during the next 24 h. On the third day of incubation IC_50_ slightly increased. Similar pattern with higher IC_50_ concentrations can be observed in MCF-7 (human breast carcinoma cell line) cells. The least sensitive cells towards BTE were cells A549 (human adenocarcinoma alveolar cell line). We observed no antiproliferative effects of BTE on mouse healthy fibroblast cell line NIH-3T3 during 72 h of influence at BTE concentrations 0.00078 – 5 μg/mL.

**Table 1:** **Cytotoxic/antiproliferative effect of BTE against HT-29, MCF-7, A549 and NIH-3T3 cell lines represented by IC**_**50**_ **(μg/mL)**

	**Single administration of BTE**			**Repetitive administration of BTE**				
	**24 h**	**48 h**	**72 h**	**24 h**	**48 h**	**72 h**	**144 h**	**216 h**
**HT-29**	<0.00078	<0.00078	0.0014	<0.00078	0.0051	0.0013	0.015	0.0125
**MCF-7**	0.0125	0.0145	0.0016	-	-	-	-	-
**A549**	>5	>5	>5	-	-	-		-
**NIH-3T3**	>5	>5	>5	-	-	-	-	-

The values represent mean ± SD of three independent experiments.

### Cytotoxic effects of BTE - repetitive administration of BTE

Because HT-29 cells were the most sensitive to the extract we have performed further studies to determine its cytotoxic effect on HT-29 cells after repetitive administration of BTE during 216 h incubation. We added BTE to the cells every 24 h (repetitive administration of BTE) during the first 3-days of incubation and then every 72 h and we counted cells at the same time points. We found no significant difference between IC_50_ during single BTE administration and repetitive administration. With increasing time of incubation cells became more resistant to the administered BTE extract.

### Type of cells death induced by BTE

**Figure 1:**
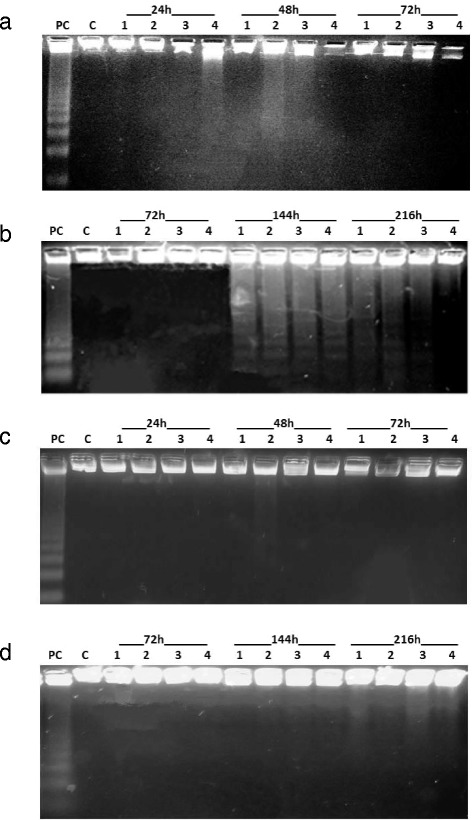
**Agarose gel electrophoresis in cells HT-29 (a, b) and MCF-7 (c, d) treated with 1–0.00625 μg/mL; 2 – 0.0125 μg/mL; 3 – 0.025 μg/mL; 4 – 5 μg/mL of BTE for 24, 48, 72, 144 and 216 h.** C – control cells without BTE, PC – positive control (L1210 cells treated with 6 μmol/L cisPt).

Analysis of DNA fragmentation was performed using conventional agarose gel electrophoresis to distinguish between apoptotic cell death and other types of cell death. Cells were exposed to single administration of BTE (0.00625 - 5 μg/mL) for 72 h and repetitive administration of BTE for 216 h of incubation. In cells HT-29 we observed no apoptotic cell death during single administration of BTE but we detected apoptosis during repetitive administration of BTE after 144 and 216 h of influence (Figure 1 a,b).

In MCF-7 cells no apoptotic cell death was detected during single and repetitive administration of BTE (Figure 1 c,d).

### Genotoxic effect

**Figure 2:**
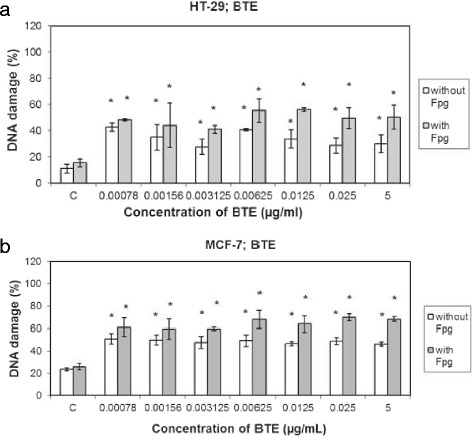
**Effects of BTE on the DNA damage (%) in HT-29 (a) and MCF-7 (b) cells after 24 h treatment.** DNA damage was represented by DNA strand breaks (without Fpg) and as the total DNA damage (with Fpg). Each value represents the arithmetic mean ± SD of three separate experiments (*n* = 3): * < 0.05 when compared with controls (HT-29 or MCF-7 cells without BTE). Maximal DNA damage (100%) for 100 comets was considered the DNA damage (TD) with the maximal score (400).

We examined the DNA damage induced by BTE (0.00078 - 5 μg/mL) after 24 h of incubation. Induction of DNA strand breaks (without Fpg) and total DNA damage (with Fpg) was dose independent in both cell lines (Figure 2a,b) and percentage of DNA strand breaks was approximately the same for each concentration used (up to 55% of DNA damage). Level of total DNA damage was higher (up to 60%). HT-29 cells were less sensitive to DNA damage compared with MCF-7 cells.

### Detection of hydrogen peroxide levels

We have determined levels of hydrogen peroxide in the culture medium of cells incubated with BTE extract (IC_50_) for up to 72 hours. At the BTE concentrations used in our experiment we have not detected any production of hydrogen peroxide in the medium. We repeated our experiments with addition of catalase (50 U/mL) to the cancer cells during 72 h of incubation with BTE extract and we did not detect any changes of hydrogen peroxide levels with or without addition of catalase.

## Discussion

White tea, yellow tea, green tea, oolong, pu-erh tea and black tea are all harvested from the same species *Camellia sinensis*, but are processed differently to attain different levels of oxidation. Fermentation of crushed tea leaves results in the production of the black tea. This process is characterized by enzymatic oxidation and condensation of polyphenolic compounds generating oligomeric (theaflavins) and polymeric (thearubigins) polyphenols (Ju et al. 2007). Their beneficial effects on health are ascribed to their antioxidant and biomodulating properties. It has been reported that theaflavins are able to inhibit certain types of cancer (Kosińska and Andlauer 2004; Tu et al. 2004; Kundu et al. 2005; Ju et al. 2007; Dvořáková et al. 2010; John et al. 2014; Korir et al. 2014).

In this study we have investigated cytotoxic/antiproliferative effects of BTE from *Camellia sinensis* on different types of carcinoma cell lines (HT-29, MCF-7, A549) and compared with effects on the healthy cell line NIH-3T3. We have found dose- and time-dependent cytotoxic effects against the carcinoma cell lines. The cell lines HT-29 (colon cancer) and MCF-7 (breast cancer) were more sensitive to the extract than A549 (lung cancer) after 72 h of cultivation. The effect of BTE on healthy cells NIH-3T3 was milder. Growth inhibitory activities of BTE in cancer cells were observed also in other studies (Sharangi 2009; Miloševič et al. 2013) with effects depending on the cell type and concentrations used.

We have studied induction of apoptosis by BTE using DNA fragmentation assay. Cells exposed to BTE exhibited characteristic internucleosomal DNA fragmentation. We used single and repetitive administrations of BTE to carcinoma cell lines HT-29 and MCF-7. We have detected apoptosis only in cells HT-29 during repetitive administration of BTE. Single BTE administration did not result in apoptosis either in HT-29 or in MCF-7 cancer cells. Contrary to our results, Yang et al. (Yang et al. 1998) found apoptosis in HT-29 cells exposed to theaflavins for 24 h. They determined apoptosis by the annexin V apoptosis assay. Several other studies have shown that *Camellia sinensis* extract induced apoptosis in cancer cell lines (Yang et al. 1998; Sun et al. 2013; Brizuela and Cuvillier 2014; Wanga et al. 2014). However, they used either much higher concentrations (more than 20x) of the BTE than we did, or different carcinoma cell lines. Prasad et al. (Prasad et al. 2007) studied the mechanism of theaflavins’ action on cellular proliferation and cell death in the human prostate cancer cell line PC-3. They found that theaflavins act as anti-proliferative agents by modulating cell growth regulators in prostate cancer cells.

In our study we have detected genotoxic effects of BTE on human carcinoma cell lines (HT-29 and MCF-7) after 24 h cultivation but this effect was dose independent. Similar results were reported also in other studies (Saha and Das 2002; Prasad et al. 2007; Halder et al. 2008; Nagini and Murugan 2013; Forbes-Hernández et al. 2014; Hajiaghaalipour et al. 2015). Polyphenolic compounds in the tea are considered strong antioxidants preventing tumor development through its antioxidant function (Feng et al. 2002; Higdon and Frei 2003). However, results of our study indicate that at high concentrations bioactive compounds in the BTE are themselves capable of oxidative damage to DNA in cancer cells probably through their pro-oxidant action. Polyphenolic compounds are redox-sensitive compounds able to reduce redox active metal ions (Fe^3+^, Cu^2+^) which can then catalyze formation of reactive hydroxyl radical (^.^OH) through the Fenton type reaction (Hadi et al. 2000; Malik et al. 2003; Forester and Lambert 2014; Tao and Lambert 2014).

## Conclusion

Our results confirmed that BTE possess potent antiproliferative and genotoxic properties against human colon carcinoma and human breast cancer cell line. Our findings indicate that BTE has the potential to become a safe therapeutic anti-carcinogenic agent. However, to support the hypothesis that black tea extract might play a role in the cancer prevention, some randomized controlled trials as well as large prospective cohort studies are needed.

## Materials and methods

### Chemicals

Normal melting point agarose (NMP agarose), Triton-X 100, ethidium bromide (EtBr), RNase, proteinase K, phosphate-buffered saline (PBSa), all culture media, fetal calf serum, antibiotics, catalase and Fpg enzyme were purchased from Sigma Aldrich (Slovakia). Low melting point agarose (LMP) was purchased from Invitrogen (Great Britain). Ethylenediaminetetraacetic acid (EDTA), ethylenediaminetetraacetic acid disodium salt (Na_2_EDTA), and sodium hydroxide (NaOH) were obtained from Lachema (Brno, Czech Republic). Kit OxiSelect™ hydrogen peroxide assay kit, Colorimetric was obtained from Cell Biolabs, Inc. (USA).

### Black tea extract

Extract from *Camellia sinensis* (black tea) (BTE) was kindly provided by Dipl. Ing. Ján Keresteš (Molecule of Life Ltd., Senec, Slovakia). The extract is water soluble. It contains active flavonoids min. 96% such as catechins (60 - 75%), leukoantocyanidins (25 - 40%), cyanidins and general flavone derivatives of diols. The complexes of flavonoids are present in mono and oligomeric form.

### Cell cultures

Human colon carcinoma cells (HT-29), adenocarcinoma human alveolar basal epithelial cells (A549), breast carcinoma cell line (MCF-7) and mouse embryonic fibroblast healthy cell line (NIH-3T3) were purchased from American Type Culture Collection (ATCC), Rockville, MD, USA. All cell lines were maintained in complete Dulbecco’s Modified Eagle’s Medium supplemented with 10% (vol./vol.) fetal calf serum, penicillin G (100 μg/mL) and streptomycin (100 μg/mL). All cell lines were grown at 37°C in humidified 5% CO_2_ and 95% air atmosphere. The HT-29 cells were plated on the Petri dishes (diameter 60 mm) at a density of 1.5 x 10^5^ cells per mL, MCF-7 and A549 at a density of 8 x 10^4^ cells per mL and NIH-3 T3 at a density of 6.0 x 10^4^ cells per mL of medium. All cell lines were incubated for 24 h prior to experiments.

### Antiproliferative/cytotoxic effect

Cell proliferation, growth curves, cell viability and cytotoxic potential of BTE (Molecule of Life, Ltd., Senec, Slovakia) was determined by the MTT test and the trypan blue dye exclusion test. MTT test is based on the reduction of yellow soluble compound 3-[4,5-dimethylthiazol-2-yl]-2,5-diphenyl tetrazolium bromide (MTT, Sigma-Aldrich Chemical, Taufkirchen, Germany) into an insoluble formazan (blue star-shaped crystals). The reaction takes place in the mitochondrial membrane of living cells. Formazan was dissolved by addition of a strong detergent and color was read spectrophotometrically at a wavelength of 540 nm. Absorbance of the solution is proportional to the number of living cells. Before reading absorbances we washed cells with PBS thus removing the interference of the polyphenols with MTT assay. By the trypan blue dye exclusion test the cell proliferation was determined by direct counting of cells in a counting chamber.

For the 3 day experiments cells A549, HT-29, MCF-7 and NIH-3T3 were seeded in 60 mm Petri dishes and after 24 h of incubation at 37°C the cells were/were not exposed to BTE for 24, 48, 72, 144 and 216 h. Growth curves, cytotoxic/antiproliferative effects and inhibitory concentrations IC_50_ (the median concentration that causes 50% inhibition of growth) were determined. All tests were performed three times. Final concentrations of BTE added to the cells were within the concentration range 0.00078 - 5000 μg/mL.

### Electrophoretic analysis

Control cells (without BTE) HT-29, MCF-7 and cells treated with BTE (final concentration of BTE was from 0.00625 to 5 μg/L) were incubated for 24, 48, 72, 144 and 216 h then harvested, washed with PBS, and then lysed in 50 μL of lysing solution (10 mmol/L Tris, 10 mmol/L EDTA, 0.5% Triton X-100) supplemented with proteinase K (1 mg/mL). Samples were then incubated at 37°C for 1 h and heated at 70°C for 10 min. Following lysis, RNase (200 μg/mL) was added and incubated at 37°C for 1 h. The cell lysates were loaded into 2% agarose gel, stained with ethidium bromide (1 mg/mL) and subjected to electrophoresis (40 V, 3 h). Separated DNA fragments (DNA ladders) were visualised on a UV transilluminator (254 nm, Ultra-Lum Electronic UV Transilluminator, USA).

### Comet assay

DNA strand breaks and oxidative DNA damage were measured using the alkaline comet assay (Collins et al. 1993). Cells were resuspended in 400 μL of 0.8% low melting point agarose in PBS at 37°C and pipetted onto a frosted microscope slide precoated with 100 μL of 1% normal melting point agarose. Slides with layers of cells in agarose were incubated in a refrigerator for 10 min (4°C), then immersed in lysis solution (2.5 mol/L NaCl, 100 mmol/L, Na_2_EDTA, 10 mmol/L Tris, 1% Triton, pH 10) for 1 h to remove cell membranes. After lysis, slides were placed in a horizontal electrophoresis tank containing electrophoresis solution (1 mmol/L Na_2_EDTA, 300 mmol/L NaOH, pH 13) at 4°C for 40 min (DNA uncoiling). Electrophoresis was performed in the same solution at 25 V, 300 mA, 4°C, for 30 min. The slides were washed three times for 5 min at 4°C with neutralising buffer (0.4 mmol/L Tris, pH 7.5) before staining with 20 μL of 4’,6-diamidine-2-phenylindole dihydrochlorid (DAPI, 1 μg/mL solution in distilled water). To detect total DNA damage (DNA strand breaks and oxidative DNA damage) we used formamidopyrimidine DNA glycosylase enzyme (Fpg). Comets were viewed by fluorescence microscopy after staining with DAPI. The intensity of the comet tail relative to the head reflects the number of DNA breaks by manual scoring. One hundred cells from each of two replicate slides were analyzed for each BTE concentration and classified into five classes according to the relative intensity of fluorescence in the tail and given a value of 0–4 (from undamaged 0, to maximally damaged 4). The total score for 100 comets could range, therefore, from 0 (all undamaged) to 400 (all maximally damaged).

Total DNAdamage (TD) was obtained by summation (Collins et al. 1993; Collins et al. 1997):

$$ \mathrm{T}\mathrm{D}={\displaystyle \sum_{\mathrm{i}=0}^4\mathrm{i}.{\mathrm{N}}_{\mathrm{i}}} $$

Where i – class of damage, N – number of cells in the class

We calculated % of DNA damage according to the formula:

$$ \mathrm{D}\mathrm{N}\mathrm{A}\ \mathrm{damage}\ \left(\%\right) = \left(\mathrm{T}\mathrm{D}/400\right)*100 $$

### Detection of hydrogen peroxide levels

Hydrogen peroxide levels in medium of cells incubated with our BTE extract with/without addition of catalase (50U/mL) for up to 72 hours were detected by the OxiSelect™ hydrogen peroxide assay kit, Colorimetric, Cell Biolabs, Inc. As a possitive control we used the sample with hydrogene peroxide (0.8 μmol/L) added in the medium.

### Statistical analysis

Results are expressed as arithmetic means ± standard deviation (SD) of the means of three separate experiments (each experiment was done with three parallels).

The statistical evaluation was performed using parametric unpaired *t*-test. A difference at P < 0.05 was considered statistically significant.

## Abbreviations

BTE: Black tea extract
DAPI: 4′,6-diamidine-2-phenylindole dihydrochlorid
Fpg: Formamidopyrimidine DNA glycosylase
IC_50_: Half maximal inhibitory concentration
PBS: Phosphate-buffered saline
PC: Positive control
TD: Total DNA damage
